# Supplementary material for: False-Positive Diagnosis of Congenital Heart Defects at First-Trimester Ultrasound: An Italian Multicentric Study
Source: Diagnostics (Basel). 2024 Nov 13;14(22):2543. doi: 10.3390/diagnostics14222543 (PMC11592638; doi:10.3390/diagnostics14222543)
Supplement: Supplementary file 1 [file diagnostics-14-02543-s001.zip › diagnostics-3240060-supplementary/Supplementary material.pdf]

Supplementary Table S1. Included cases of congenital heart disease suspected in the first trimester.

#	Suspected CHD at the first trimester scan	GA	FE	GA	Post-natal or pot-mortem confirmation	Outcome	Genetic results	NT (mm)	Screening risk	Extracardiac abnormalities
**1**	AVSD	12^{+0}	Complete AVSD	16^{+0}	Yes, postmortem	TOP	Trisomy 21	4	High risk	No
**2**	AVSD	11^{+1}	Complete AVSD	12^{+1}	Yes, postmortem	TOP	Normal	6	High risk	Exomphalos, sirenomelia, cleft palate, SUA
**3**	AVSD+ ARSA	12^{+0}	Complete AVSD	12^{+4}	Yes, postmortem	TOP	Trisomy 21	4,6	ND	Absent nasal bone
**4**	AVSD	13^{+2}	Complete AVSD	16^{+1}	Yes, postmortem	TOP	Trisomy 21	2,3	High risk	No
**5**	AVSD	13^{+6}	Normal	18^{+0}	Yes, postnatally	Livebirth	Normal	2,8	High risk	No
**6**	HLSH	12^{+0}	HLSH	16^{+0}	Yes, postmortem	TOP	Normal	5	High risk	Bilateral hydronephrosis, SUA
**7**	HLHS	12^{+4}	HLHS	15^{+6}	Yes, postmortem	TOP	Normal	1,80	Low risk	No
**8**	HLHS	13^{+6}	HLHS	14^{+0}	Yes, postmortem	TOP	N.D	1,2	Low risk	Spina bifida
**9**	HLHS	13^{+4}	HLHS	19^{+2}	Yes, postmortem	TOP	Monosomy X	13,6	ND	Fetal hydrop
**10**	HLHS	12^{+1}	HLHS	18^{+6}	Yes, postmortem	TOP	46 XX microdel16	1,1	Low risk	No
**11**	Ventricular dysproportion (LV<RV)	12^{+0}	HLHS	18^{+3}	Yes, postmortem	TOP	Trisomy 18	1,4	High risk	Ectrodactyly
**12**	Ventricular dysproportion (LV>RV)	13^{+5}	Tricuspid atresia+VSD, TGA	16^{+3}	Yes, postmortem	TOP	Normal	6	High risk	No
**13**	Ventricular dysproportion (LV<RV)	12^{+5}	CoA	15^{+2}	Yes, postmortem	TOP	Trisomy 21	3	High risk	Exomphalos
**14**	CoA	12^{+3}	CoA	16^{+2}	Yes, postnatally	Livebirth	Monosomy X	7,50	High risk	CPF
**15**	DORV	11^{+0}	DORV	13^{+2}	Yes, postmortem	TOP	46 XX microdup16p13.11	4,2	Low risk	No

**16**	DORV	12^{+4}	DORV	18^{+2}	Not available	Selective fetocide	Normal	1,5 e 1,6	Low risk	No
**17**	RAA	12^{+3}	RAA	20^{+3}	Yes, postnatally	Livebirth	N.D.	1,3	Low risk	No
**18**	RAA	13^{+2}	RAA+ALSA	18^{+0}	Yes, postnatally	Livebirth	Normal	2,3	High risk	No
**19**	Aortic stenosis	14^{+0}	Critical aortic stenosis	16^{+0}	Yes, postmortem	TOP	Normal	1,38	Low risk	No
**20**	Suspected cardiac defect (abnormal 4CV)	13^{+0}	AVSD	15^{+0}	Yes, postmortem	TOP	47XXX	2,1	High risk	Fetal hydrop
**21**	Suspected cardiac defect (abnormal 3VTV)	13^{+4}	Normal	18^{+0}	Yes, postnatally	Livebirth	Normal	2,7	Low risk	No
**22**	Suspected cardiac defect (abnormal 3VTV)	12^{+3}	Normal	16^{+0}	Yes, postnatally	Livebirth	N.D.	1,38	No	No

CHD, congenital heart disease; GA, gestational age; FE, fetal echocardiography; NT, nuchal translucency; AVSD, atrioventricular septal defect; TOP, termination of pregnancy; HLHS, hypoplastic left heart syndrome; SUA, single umbilical artery; CoA, coarctation of the aorta; CPF, cystic posterior fossa; LV, left ventricle; RV, right ventricle; IVSD, interventricular septal defect; TGA, transposition of the great arteries; ARSA, aberrant right subclavian artery; DORV, double outlet right ventricle; 3VTV, three vessel trachea view; RAA, right aortic arch; ALSA, aberrant left subclavian artery.

Supplementary Video S1

A case of hypoplastic left heart syndrome with reverse flow in the aortic arch in the three vessel and trachea view and filling of a single ventricle in the four-chamber view.

Supplementary Video S2

A case of atrioventricular septal defect in which the single entrance at the level of the atrioventricular valve is clearly seen with the regular filling of the two ventricles. The presence of regurgitation through the dysplastic valve is also shown.
